# Amorphous lead oxide (a-PbO): suppression of signal lag via engineering of the layer structure

**DOI:** 10.1038/s41598-017-13697-2

**Published:** 2017-10-16

**Authors:** O. Semeniuk, O. Grynko, G. Juska, A. Reznik

**Affiliations:** 1 Chemistry and materials science program, Lakehead University, 955 Oliver Road, Thunder Bay, ON P7B 5E1 Canada; 2 Advanced detection devices department, Thunder Bay Regional Health Research Institute, 290 Munro Street, Thunder Bay, ON P7A 7T1 Canada; 3 Department of Solid State Electronics, Vilnius University, Saulėtekio 9 III k., 10222 Vilnius, Lithuania; 4 Department of Physics, Lakehead University, 955 Oliver Road, Thunder Bay, ON P7B 5E1 Canada

## Abstract

Presence of a signal lag is a bottle neck of performance for many non-crystalline materials, considered for dynamic radiation sensing. Due to inadequate lag-related temporal performance, polycrystalline layers of CdZnTe, PbI_2_, HgI_2_ and PbO are not practically utilized, despite their superior X-ray sensitivity and low production cost (even for large area detectors). In the current manuscript, we show that a technological step to replace nonhomogeneous disorder in polycrystalline PbO with homogeneous amorphous PbO structure suppresses signal lag and improves time response to X-ray irradiation. In addition, the newly developed amorphous lead oxide (a-PbO) possesses superior X-ray sensitivity in terms of electron-hole pair creation energy ${W}_{\pm }$ in comparison with amorphous selenium – currently the only photoconductor used as an X-ray-to-charge transducer in the state-of-the-art direct conversion X-ray medical imaging systems. The proposed advances of the deposition process are low cost, easy to implement and with certain customization might potentially be applied to other materials, thus paving the way to their wide-range commercial use.

## Introduction

Amorphous and polycrystalline modifications of wide band gap semiconductors are of paramount importance in modern electronics, since they allow large device area production at low cost. However, the transition from crystalline to non-crystalline materials is technologically challenging since structural disorder may lead to degradation of the material performance. A good example is CdTe and CdZnTe (CZT): the combination of outstanding photoconductive properties, and high atomic number of these materials made them widely employed in sensing electromagnetic radiation. Particularly, CdTe and CdZnTe play a major role in X-ray and gamma-ray detectors for a variety of applications ranging from nuclear physics and astrophysics to homeland security and medical imaging^1–10^. However, polycrystalline forms of these materials are much less utilized. While the properties of poly-CdTe films are fit for optical photon harvesting in high efficiency solar cells^11–14^, there are reported to be challenges for applications in X-ray medical imaging. Particularly, both poly-CdTe and poly-CdZnTe suffer from a residual signal after exposure termination, called signal lag^15–18^. The presence of a residual signal limits the application of these materials to static imaging (for instance, radiography), while the needs of vitally-important real-time imaging, like fluoroscopy, are left unmet. Interestingly, other polycrystalline high-Z (atomic number) materials that have also been considered for applications as radiation detectors (including polycrystalline layers of PbI_2_, HgI_2_, and poly-PbO^19–23^) are all suffering from the same problem, i.e. signal lag. The values of the residual currents range from 10–30% at 2–5 frame per second (fps) read-out in PbI_2_ and HgI_2_^20,21,23^ to 4–9% at 1 fps in poly-PbO^19^, meaning that a significant fraction of the X-ray generated charge is collected after X-ray exposure is terminated. The similarity in X-ray response might be linked to a common feature of their layer structure: all these materials are highly inhomogeneous and are composed of grains which are 1–3 μm in size for PbI_2_ and PbO^19,20^, and 30–60 μm for high-quality HgI_2_ layers^20^. It was suggested that the lag in these materials is caused by the presence of grain boundaries and associated defects which act as localized trapping sites for X-ray generated charge^20–23^. In addition, in poly-PbO lag is influenced by charge *injection* from the bias electrodes^19,24^. Indeed, it was shown that in poly-PbO the magnitude of lag depends on the material used for the bias electrodes and is significantly suppressed for operation with electron beam-read out^25–27^.

In terms of signal lag, the only exemption in the series of disordered materials considered for application in radiation sensing, is amorphous selenium (a-Se), where technological advances allowed suppression of signal lag to a level that this material became a practical solution for advanced direct conversion X-ray medical imaging detectors. Due to the comparatively low-Z of a-Se, its properties are well suited for applications in mammography energy range (20–30 keV)^28,29^. However, for general radiographic and fluoroscopic applications a-Se has to be replaced by a lag-free higher Z material. Interestingly, in contrast to the high-Z polycrystalline materials mentioned above, a-Se has a *uniform* and homogeneous layer structure, composed of chains and rings of Se atoms^30,31^ without grain boundaries^32^. This suggests a possible approach to combat signal lag in disordered photoconductors, namely, to develop a grain boundary-free structure for lag-free operation.

In the current manuscript, we evaluate the image lag and X-ray sensitivity in the newly developed homogeneous amorphous PbO structures. We show that elimination of the PbO polycrystalline structure has a very peculiar effect on the X-ray performance of PbO: signal lag was significantly improved, while X-ray sensitivity remains higher than reported for a-Se. The measurements performed at various exposures and at an extended range of electric fields suggests the suitability of a-PbO for real time imaging at 30 frames per second (fps).

## Results

### Temporal behavior

**Figure 1:**
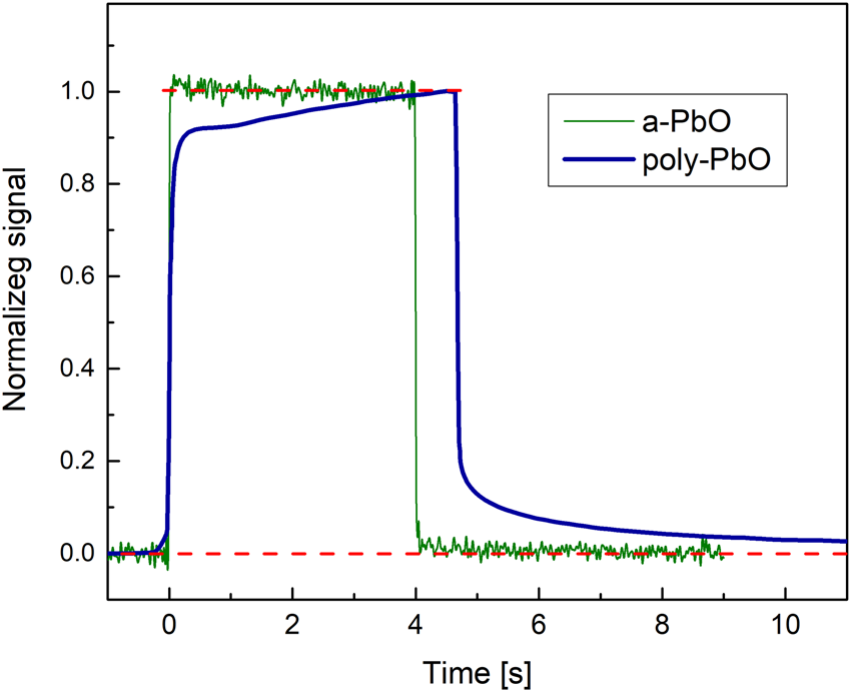
The response of a-PbO at *F* = 10 V/μm to 4 s X-ray exposure is compared with previously reported data on poly-PbO, replotted from Fig. 4 in ref.^19^. The X-ray signal was offset corrected for the dark current. For this purpose, the average of data points before exposure was subtracted from the X-ray trace of a-PbO.

Figure 1 shows the typical response of an a-PbO detector to a 4 s X-ray pulse. The amplitude of the signal remains constant during the exposure, while after termination of X-rays it promptly drops to the dark current level. Figure 1 also shows results obtained with polycrystalline PbO layers, which were grown by the conventional thermal evaporation technique and consist of a network of crystalline platelets as described in ref.^19^. Despite the measurements were performed under similar experimental condition (similar X-ray pulse duration and energy range) there is a pronounced difference in the X-ray response. Indeed, poly-PbO exhibits a signal build-up during the exposure, followed by a relatively long lag.

**Figure 2:**
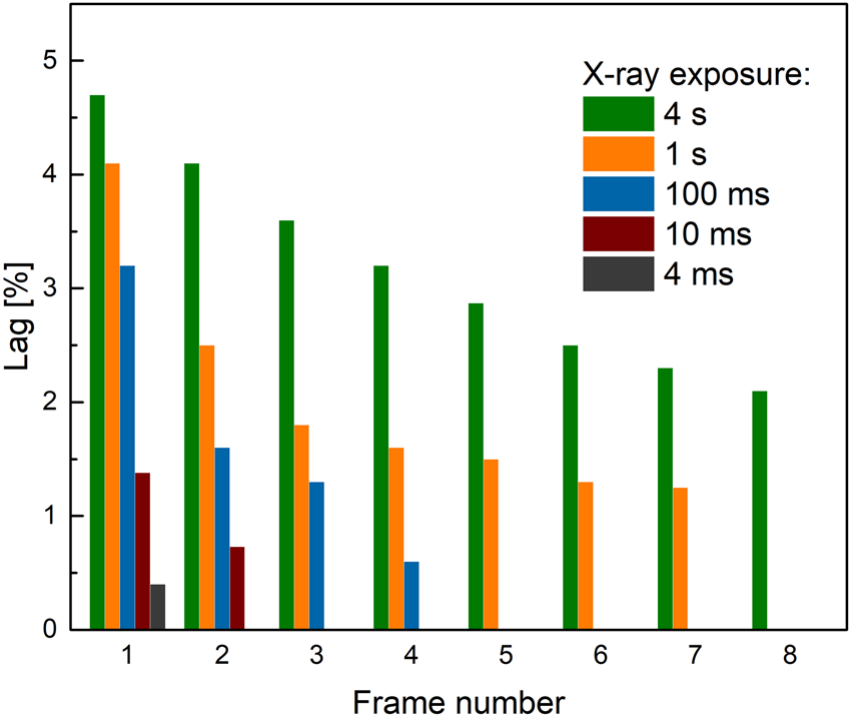
The lag measured at read-out rate of 30 fps in a-PbO at 10 V/μm is shown for different X-ray pulses.

Figure 2 shows signal lag of a-PbO detector measured under various experimental conditions, relevant to those used in pulsed fluoroscopy. For this purpose, the detector was exposed to a short (4 ms and 10 ms) X-ray pulse and an X-ray response was readout every 33 ms (i.e. at 30 fps)^33,34^. The lag was determined as the ratio of the residual signal after exposure to the X-ray signal value. For example, following the convention described in refs^33,34^, the residual signal after the first frame i.e. in 33 ms after termination of exposure, was found to be ~1.4% for 10 ms X-ray pulse. The signal lag during consequent frames was measured with 33 ms intervals showing the lag values <1% of the signal magnitude during the exposure. As seen from Fig. 2, signal lag of a-PbO increases with exposure duration, which was previously observed in other materials, like a-Se and poly-PbO^34,35^. Thus, the first frame lag of a-PbO was found to be 0.4% and 1.4% after single X-ray pulses of 4 ms and 10 ms, respectively. The lag dropped to undetectable values after the first frame for the shortest pulse duration (4 ms) and after the second frame for 10 ms pulse. While the data obtained compares favorably with lag measured in a-Se detectors of 1.5% and 3.7% for same pulse durations^33,36,37^, it is also important to check the residual signal under longer exposures, which are more relevant to fluoroscopic imaging. To simulate such conditions, a-PbO detector was subjected to extended exposures of 100 ms, 1 s and 4 s. The corresponding values for the first frame lag are: 3.2%, 4.1% and 4.7%, respectively. The obtained values are also comparable with those measured on a-Se direct conversion and CsI indirect conversion detectors, which normally exhibit the first frame lag values less than 10% and 5%, respectively^34,35,38–40^. It should be noted that lag in these detectors was measured in the pulsed fluoroscopy mode, i.e. with sequence of short exposures 1–8 ms every 33 ms so that detector material has time to “rest” and recover between exposures. Nevertheless, as this is seen from Fig. 2, with the subsequent frames the lag quickly drops even for long exposures; it is no longer detected after the fourth frame for 100 ms pulse duration and after the eighth frame for 1 s pulse. For the longest pulse of 4 s the lag is still at ~2% after eight frames, although this long pulse of X-rays represents extreme operation conditions with extra load on the detector. It should be noted, that our preliminary investigation showed no effect of various dose rates on the temporal response i.e. lag magnitude and its kinetics.

**Figure 3:**
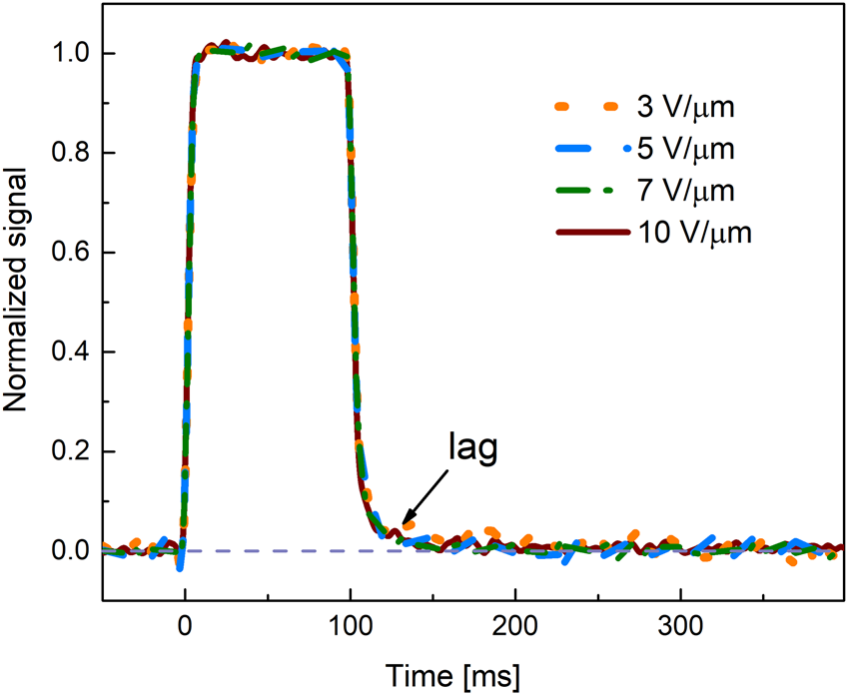
The normalized response of a-PbO to 100 ms X-ray pulse.

Figure 3 illustrates normalized response of a-PbO to 100 ms exposures measured at selected electric fields. Residual signal is shown to be scalable with applied electric field, i.e. no lag improvement is observed at higher fields.

### X-ray sensitivity

**Figure 4:**
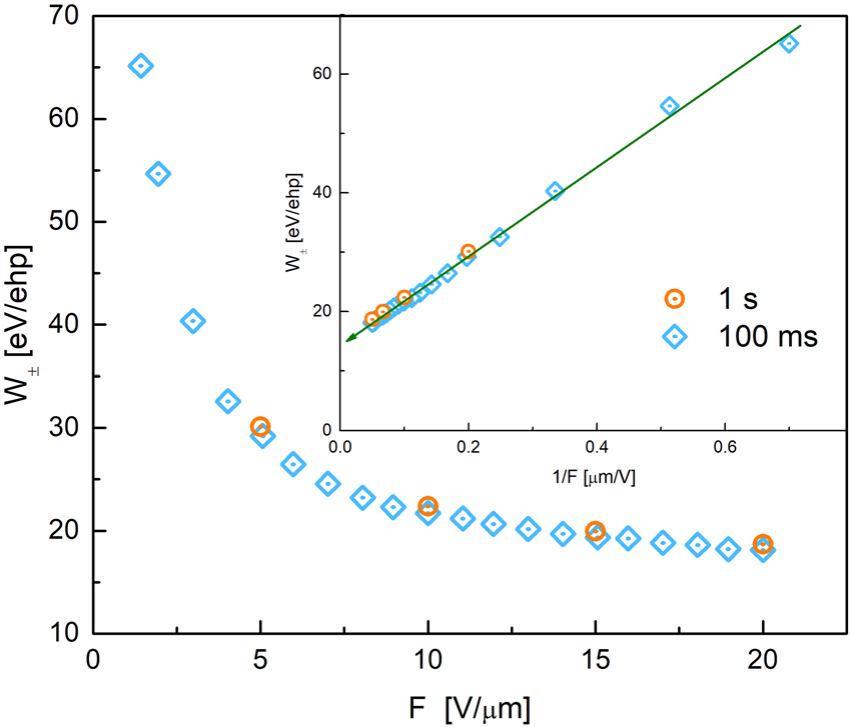
Measured values of ${W}_{\pm }$ are plotted as a function of $F$ for 100 ms and 1 s exposures. The inset to the figure shows ${W}_{\pm }$ plotted as a function of $1/F$.

The sensitivity of a-PbO to X-rays is characterized in terms of energy, required for creation of a single electron-hole pair ${W}_{\pm }$. This quantity was derived from the total charge collected with a-PbO detector upon X-ray exposure. More details on this analysis can be found elsewhere^19,24,41^. The measurements of the X-ray response of a-PbO performed at different electric fields $F$ show that ${W}_{\pm }$ decreases with $F$ as shown in Fig. 4, reaching the value of 22 eV/ehp and 18 eV/ehp at 10 V/μm and 20 V/μm, respectively. The measurements performed at 100 ms and 1 s X-ray pulses provide essentially the same values within ~3%. Plotting ${W}_{\pm }$ as a function of inverse field and extrapolating it (with a linear fit) to the infinite field reveals saturation at ~14 eV/ehp (see inset to Fig. 4).

In the Fig. 4 we show the dependence of ${W}_{\pm }$, calculated from X-ray photocurrent response, on electric field $F$ for 100 ms and 1 s X-ray pulse durations. While the dose rate is the same for both measurements (~2 Roentgens per second), the longer expose time results in the higher total dose delivered to the detector. Thus, Fig. 4 also provides information on the dependence of ${W}_{\pm }$, on the dosage: ${W}_{\pm }$ in a-PbO remains the same for different X-ray doses, used within our experiments.

## Discussion

If a photoconductor is considered for applications in real-time i.e. fluoroscopic, imaging, its temporal performance (in terms of the signal rise time under X-ray exposure and the signal fall time once the exposure is terminated) is of particular importance. Indeed, fluoroscopic imaging is the most challenging and demanding radiation medical procedure, since the detector is exposed to very short, 2–4 ms (sometimes 1–10 ms), X-ray pulses at ~70 kVp, while the image acquisition rate is very fast: 30 frames per second (fps)^33,40,42^. Such dynamic read out is needed to capture the motion of the human structures and requires very little residual signal at the end of each frame, otherwise the previous image will be superimposed on the subsequent ones, resulting in a misleading view. Therefore, a quasi-rectangular shape of X-ray generated current and the absence of lag are important figures of merit to evaluate the readiness of an X-ray-to-charge transducer for direct conversion X-ray medical imaging detectors. Figures 1 and 2 demonstrate the significant signal lag suppression in a-PbO in comparison with its polycrystalline form: for the most relevant to fluoroscopic procedures pulse duration of 4 ms, the first frame lag was as small as 0.4% and was undetectable after the second frame. Even for the extended exposures the observed lag never exceeded 5%, thus making the temporal performance of a-PbO comparable with CsI detectors, which are currently in use for fluoroscopic applications.

When it is detectable, the lag in a-PbO has an interesting behavior: it depends on the exposure duration, while it is scalable with the applied electric field (see Figs 2, 3). Similar behavior was observed on a-Se layers and was related to electronic processes at the bias electrode interfaces, which facilitate injection^34^. The analogy with a-Se suggests injection as the primary cause for lag in a-PbO. Such injection builds-up during exposure^43,44^ and interferes with X-ray sensitivity measurements, and misleadingly reducing ${W}_{\pm }$ values at longer X-ray pulse durations^24^. However, ${W}_{\pm }$ measurements performed on a-PbO (see Fig. 4) remain within 3% of the measured values with increase in pulse duration by a factor of 10, indicating an insignificant contribution of X-ray-modulated injection on ${W}_{\pm }$ measurements in a-PbO. The obtained ${W}_{\pm }$ values compare very favourably with those reported for a-Se: at $F$ = 10 V/μm, ${W}_{\pm }$ of a-PbO is ~22 eV/ehp, which is about a half the a-Se value, measured at the same electric field^45^.

Overall, our findings suggest an interesting and non-obvious approach to the improvement of X-ray response: replace the spatial disorder of grain boundaries in polycrystalline films with uniform disorder in amorphous layers.

**Figure 5:**
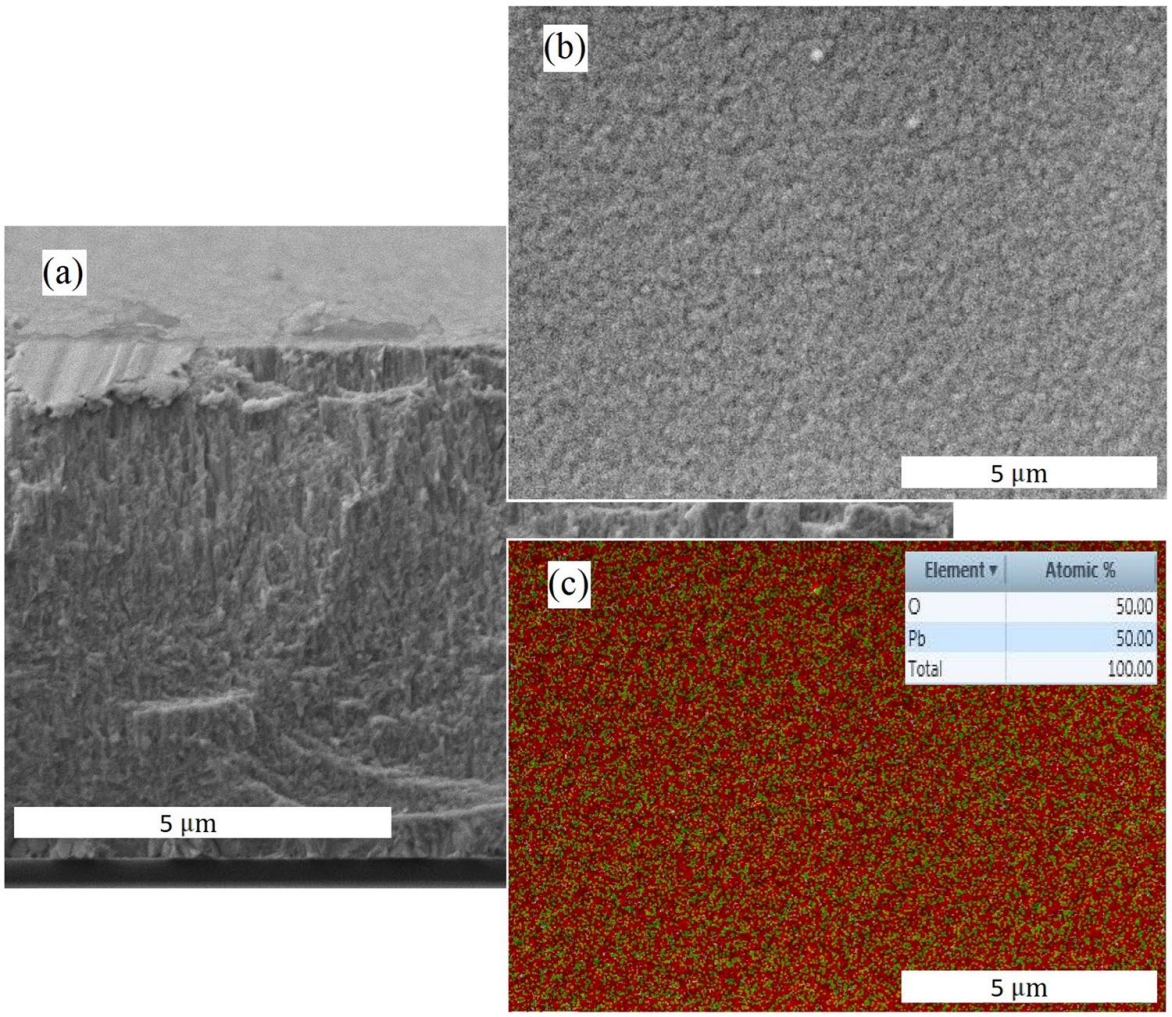
Morphological analysis of a-PbO samples: (**a**) SEM of a-PbO cross-section; (**b**) SEM micrograph of the a-PbO surface; (**c**) EDS of the surface. Red color represents Pb atoms and green color is for oxygen. It should be noted that the signal from Pb is stronger than that from O (typical for EDS measurements), therefore picture looks more red, while material is perfectly stoichiometric^46^.

Indeed, morphological analysis performed with scanning electron microscopy (SEM) (see Fig. 5), indicates that a-PbO layers, deposited with ion assistance, are uniform and free of the platelets, which appear when poly-PbO is deposited with basic thermal evaporation technique^19^.

Such a structural transformation facilitates significant lag improvement in PbO, while preserving its high X-ray sensitivity (i.e. low ${W}_{\pm }$). In addition, the deposition process behind this structural transformation, also results in a stoichiometric layer, thus solving the issue of oxygen deficiency – a common problem of metal oxides.

Finally, we would like to note that the proposed low-cost optimization of the deposition process which was applied to PbO can potentially be applied (with certain customization of course) to other materials, like PbI_2_, HgI_2_ and CZT. Since, these materials possess the similar layer structure and suffer from similar artefacts, the advancement of the deposition process with ion bombardment and *amorphization* of the layer structure might also pave the way for many other photoconductors with practical application in commercial devices.

## Methods

The ~8 μm thick amorphous lead oxide (a-PbO) samples were grown by an ion assisted thermal evaporation technique. In this technique, high quality PbO powder (5 N) was thermally evaporated at ~1000 °C on an ITO covered glass substrate. During the deposition process, the growing PbO layer undergoes a phase transition from polycrystalline to an amorphous structure as a result of continuous bombardment of the growing layer with oxygen ions. The substrate temperature did not exceed 100–150 °C, as indicated by temperature labels installed on the back of the substrate. The details of the ion assisted evaporation processes, as well as structural and morphological characterization of poly-and a-PbO layers can be found in ref.^46^. Scanning electron microscopy (SEM) measurements show that the a-PbO grows as highly packed layer (see Fig. 5), while the bi-dimensional mapping of the energy dispersive X-ray spectroscopy (EDS) indicates a uniform distribution of lead and oxygen atoms in the sample and 1:1 stoichiometry (see inset to Fig. 5c), which also was previously reported in our earlier study^46^. For electrical measurements, a solid gold contact (1 mm in diameter) was directly deposited *eX-situ* by sputtering atop the a-PbO film in a dedicated chamber. Investigation of X-ray performance of a-PbO was performed at ambient conditions, since in contrast to poly-PbO, it was found to be stable in air, as reported in ref.^46^.

**Figure 6:**
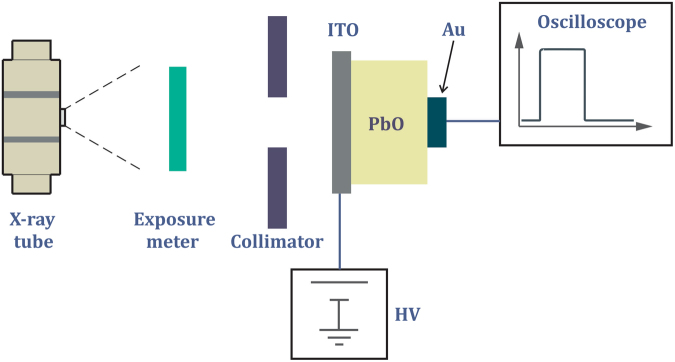
Schematic diagram of experimental apparatus for X-ray performance evaluation.

Figure 6 shows the typical experimental apparatus for X-ray performance evaluation. The X-ray tube model PX1412CS operated at 60 kVp (tube current of 100 mA for exposure time of 1 s) was used to generate X-ray pulses of various duration ranging from 4 ms to 4 seconds. A 1.5 mm thick aluminum plate was used to cut off the low energy X-rays (up to 13 keV) from entering the detector. The exposure to a-PbO layers was monitored with a Keithley model 96035 ionization chamber, which showed from 17 mR to 17 R exposure depending on X-ray pulse duration. A lead collimator 2 mm thick was used to minimize stray scattering. The model PS350 Stanford Research Systems power supply provided a constant electric field applied to the sample. During all experiments, a positive polarity was applied to the ITO. The X-ray response of the a-PbO detector was observed on the 1 MOhm input of a model TDS 420 Tektronix oscilloscope.

## Electronic supplementary material

Supplementary information
