# Supplementary material for: Amorphous lead oxide (a-PbO): suppression of signal lag via engineering of the layer structure
Source: Sci Rep. 2017 Oct 16;7:13272. doi: 10.1038/s41598-017-13697-2 (PMC5643314; doi:10.1038/s41598-017-13697-2)

Supplementary Information

Re: manuscript SREP-17-27702A " Amorphous lead oxide (a-PbO): suppression of signal lag via engineering of the layer structure" by O. Semeniuk, O. Grynko, G. Juska and A. Reznik.

Part 1:

The stray capacitance might greatly affect the measured response of the X-ray photoconductor. In order to clarify this, we deposited several poly-PbO layers with different thicknesses between 9 and 40 μm, following the recipe as described elsewhere [19] and characterized the X-ray response of poly- and a-PbO in the same measurement system. All poly-PbO layers exhibit lag. The results for 40 μm thick poly-PbO layer in comparison with a-PbO obtained using the same XPM apparatus are shown in Fig. A below. It closely resembles the Fig. 1 from the manuscript. In addition, Fig. B compares the response of our home-grown poly-PbO and poly-PbO deposited by Simon et al. (extracted from Fig. 4 in Ref. [19]). A very similar temporal performance and the same lag magnitude are evident from this comparison, suggesting that the signal lag is a feature of poly-PbO and it is present regardless of an experimental set-up used.

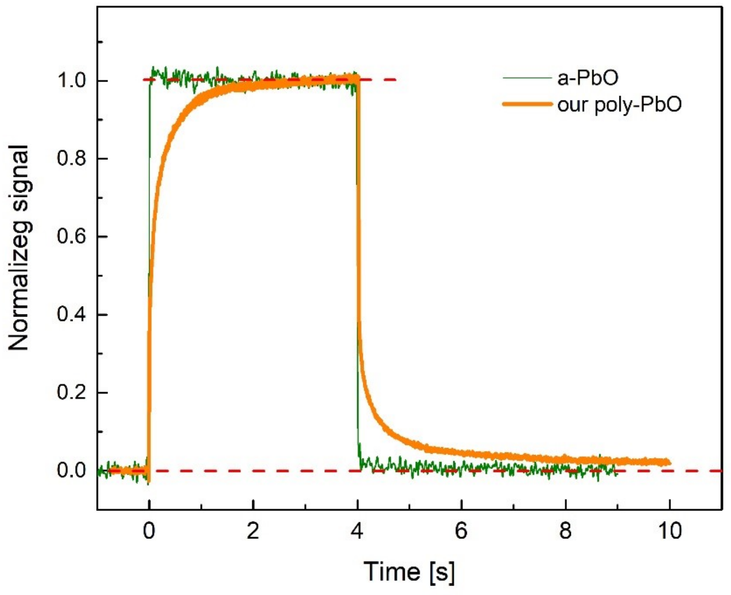

Figure A. The response of our home-grown poly-and a-PbO at F = 10 V/μm to 4 s X-ray exposure.

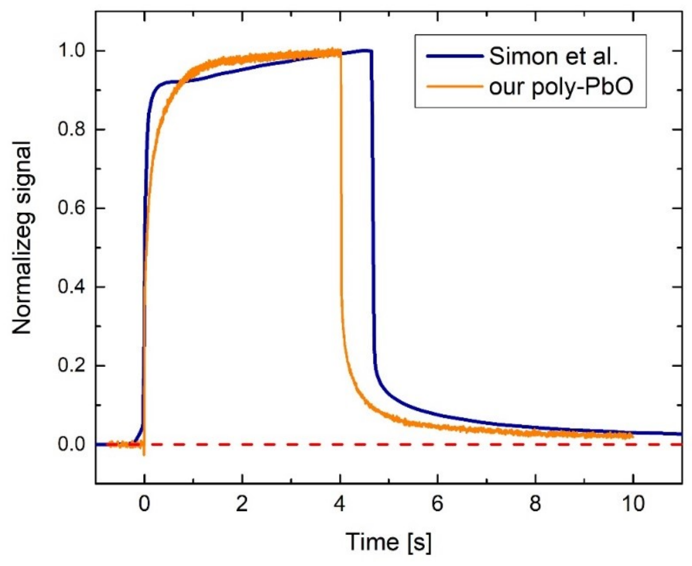

Figure B. The X-ray response of our home-grown poly-and poly-PbO by Simon et al. (extracted from Fig. 4 in Ref. [19]).

Part 2:

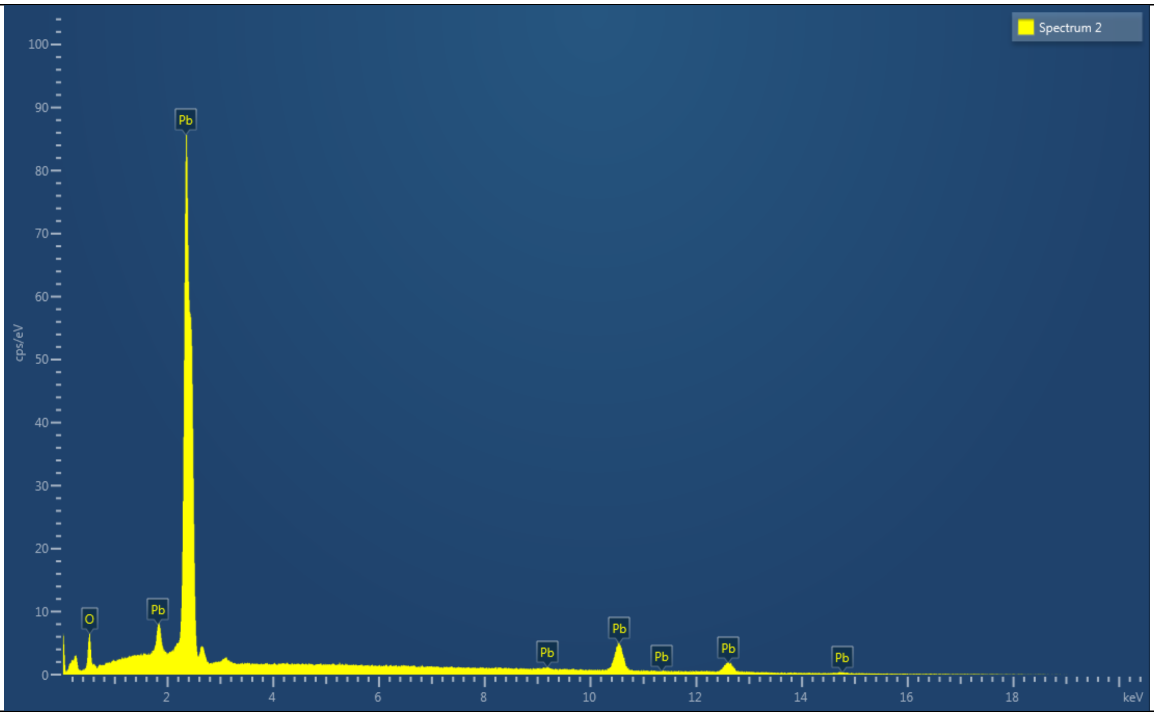

Supplement: Supplementary file 1 — Supplementary information [file 41598_2017_13697_MOESM1_ESM.pdf]
